# Case Report: Multimodal management of duodenal metastasis from hepatocellular carcinoma complicated by intractable hemorrhage

**DOI:** 10.3389/fimmu.2026.1785498

**Published:** 2026-06-03

**Authors:** Xin Zhang, Zhongsong Gao, Jiao Wang, Baiguo Xu, Bo Li, Bo Feng, Kefeng Jia

**Affiliations:** 1 Department of Interventional Radiology, Central Hospital, Tianjin University/Tianjin Third Central Hospital, Tianjin, China; 2 Department of Medical Imaging, Central Hospital, Tianjin University/Tianjin Third Central Hospital, Tianjin, China; 3 Department of Gastroenterology and Hepatology, Central Hospital, Tianjin University/Tianjin Third Central Hospital, Tianjin, China; 4 Outpatient Nursing Department, Central Hospital, Tianjin University/Tianjin Third Central Hospital, Tianjin, China

**Keywords:** gastrointestinal metastasis, hepatocellular carcinoma, immunotherapy, interventional embolization, tislelizumab

## Abstract

Hepatocellular carcinoma with duodenal involvement is a rare clinical entity with limited treatment options. Here, we report a case of advanced hepatocellular carcinoma and portal vein tumor thrombus with metastatic involvement of the duodenum that resulted in a recurrent gastrointestinal hemorrhage in a 64-year-old male. Following comprehensive diagnostic evaluation, including endoscopy, imaging, and histopathological biopsy, the patient was managed with a multimodal approach that consisted of interventional embolization for hemostasis, followed by systemic immunotherapy with tislelizumab. This therapeutic strategy successfully achieved hemorrhage control and resulted in significant radiological regression of both the duodenal metastatic lesion and the primary hepatic tumor. This case highlights the importance of a systematic diagnostic approach and suggests that immunotherapy-based regimens may hold promise in the management of advanced hepatocellular carcinoma with uncommon metastatic manifestations.

## Introduction

Hepatocellular carcinoma (HCC) is a leading cause of cancer-related mortality worldwide, and its incidence is strongly associated with hepatitis B virus infection, particularly in endemic regions such as China (1, 2). Although multimodal therapeutic approaches, such as transarterial chemoembolization (TACE), hepatic arterial infusion chemotherapy (HAIC), targeted therapy, and immunotherapy, have improved clinical outcomes in patients with advanced HCC, distant metastasis remains a principal driver of treatment failure (3). The most common metastatic sites include the lungs and lymph nodes; in contrast, gastrointestinal metastasis, especially to the duodenum, is exceedingly rare, with an incidence reported to be less than 1% in the literature (4). These metastases frequently lead to refractory upper gastrointestinal hemorrhage. Conventional endoscopic interventions are often limited in efficacy, whereas surgical resection is typically contraindicated because of an extensive tumor burden or impaired hepatic function (5). We present a case of advanced HCC with portal vein tumor thrombus (PVTT) at initial diagnosis, which later progressed to duodenal metastasis manifesting as recurrent gastrointestinal bleeding. A multidisciplinary management strategy centered on transarterial embolization successfully achieved hemostasis. Subsequent incorporation of immune checkpoint inhibitor therapy resulted in marked radiological regression of both the primary hepatic lesion and the duodenal metastatic focus. This case highlights the value of a coordinated, multimodal approach and offers insights into the potential role of immunotherapy-integrated regimens in the management of rare and challenging metastatic complications of HCC.

## Case presentation

In November 2024, a 64-year-old male patient with hematemesis and melena lasting 16 hours presented to the emergency department of our institution. His medical history included hypertension, type 2 diabetes mellitus, and chronic hepatitis B-related liver cirrhosis, for which he had been on long-term oral entecavir therapy for antiviral suppression. The patient was initially diagnosed with HCC in September 2021, following a one-month history of hematemesis. At that time, contrast-enhanced computed tomography (CECT) scans revealed findings consistent with those of HCC, including a hepatic lesion in the caudate lobe, accompanied by a tumor thrombus involving the main portal vein, its left/right branches, and intrahepatic tributaries, as well as secondary portal vein cavernous transformation (**Figures 1A–C**). Clinically, the patient was diagnosed with Barcelona Clinic Liver Cancer (BCLC) Stage C and China Liver Cancer (CNLC) Stage IIIa. Initial gastroscopy confirmed acute bleeding from the ruptured oesophagogastric varices, which was successfully controlled via endoscopic band ligation and sclerotherapy.

**Figure 1:**
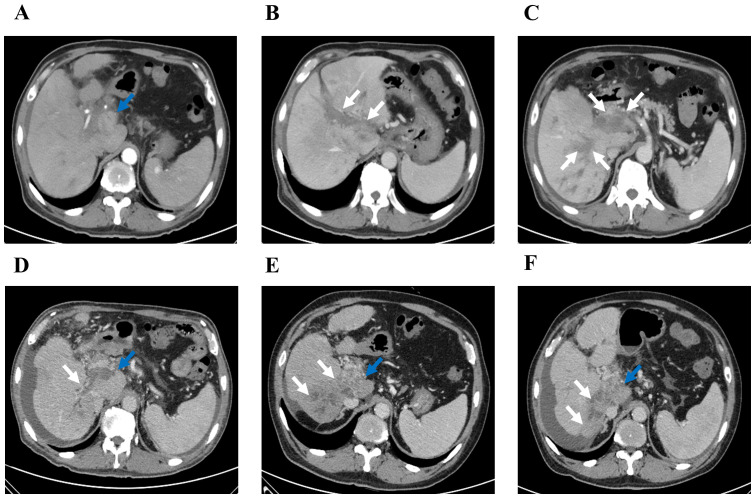
Imaging progression of intrahepatic lesions and portal vein tumor thrombus on abdominal enhanced CT. **(A)** Arterial phase image at initial diagnosis (December 2019) shows a hypervascular tumor in the hepatic caudate lobe (blue arrow); **(B, C)** Portal venous phase images at diagnosis demonstrate extensive tumor thrombus within the main portal vein and its branches (white arrow) accompanied by portal cavernous transformation; **(D)** Follow-up scan in July 2022 after treatment shows partial response (PR) of the intrahepatic lesion (blue arrow) and regression of the portal vein tumor thrombus (white arrow); **(E, F)** Subsequent scans in December 2023 and April 2024 indicate disease progression (PD) of the intrahepatic lesion (blue arrow) and re-expansion of the portal vein tumor thrombus (white arrow).

Over the subsequent 3-year treatment course, the patient underwent multiple rounds of antitumor therapy, including one session of drug-eluting bead TACE (DEB-TACE) and 11 cycles of HAIC with the FOLFOX regimen. Anticoagulation therapy was not administered in the setting of PVTT. Given the patient’s history of variceal bleeding and the malignant nature of the thrombus, anticoagulation therapy was not considered beneficial or safe. The detailed treatment timeline is presented in **Figure 2**. During this period, the intrahepatic lesions and PVTT achieved a partial response (PR) according to the modified Response Evaluation Criteria in Solid Tumours (mRECIST) and remained transiently stable but eventually progressed (**Figures 1D–F**). Serial endoscopic images (**Figures 3A–D**) revealed interval morphological changes, from a subtle mucosal elevation in March 2022 to a well-circumscribed, actively bleeding mass on April 2024 and subsequently to an enlarged lesion complicated by hemorrhage and partial duodenal obstruction by November 2024. Notably, while intrahepatic tumor progression was initially identified on endoscopy in April 2024, malignant duodenal changes did not occur (**Figure 3E**).

**Figure 2:**
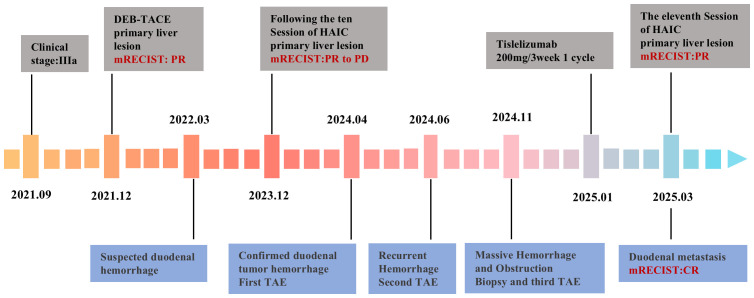
Timeline of disease course and therapeutic interventions. Response assessment for the primary lesion and PVTT was performed using modified Response Evaluation Criteria in Solid Tumors (mRECIST). After tislelizumab plus the 12th cycle of HAIC, the primary lesion and PVTT showed a partial response (PR). For the duodenal metastasis, November 2024 CECT (**Figures 4I, J**) was used as the baseline target lesion, and March 2025 CECT (**Figures 4K, L**) demonstrated a complete response (CR).

**Figure 3:**
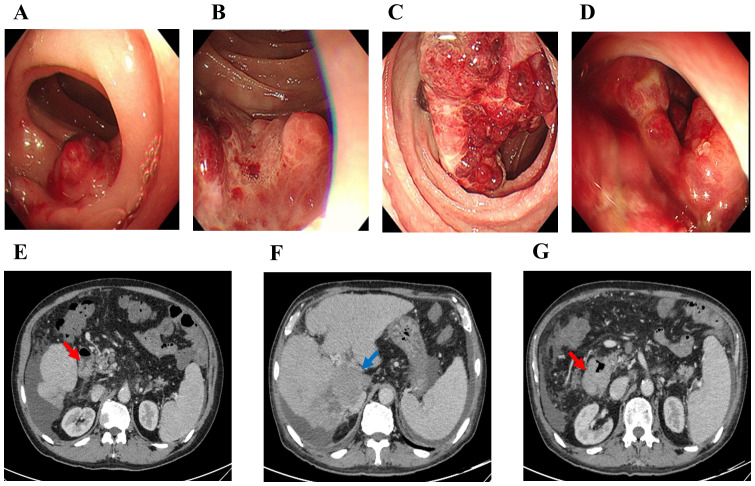
Evolution of duodenal metastasis lesions on endoscopy and CT. **(A–D)** Endoscopic images showing progressive enlargement of a mass in the descending duodenum: **(A)** March 2022, a subtle mucosal protrusion; **(B)** April 2024, a well-defined mass with active bleeding; **(C)** June 2024, further enlargement of the mass; **(D)** November 2024, the mass causing luminal narrowing and active hemorrhage; **(E)** Contrast-enhanced CT in April 2024, corresponding to the time of endoscopic bleeding **(B)**, shows no obvious duodenal wall thickening (red arrow), highlighting the superior sensitivity of endoscopy for early detection; **(F, G)** CT in November 2024 shows progression of the caudate lobe tumor (blue arrow in F) and obvious irregular thickening of the duodenal wall (red arrow in G).

To control bleeding from duodenal metastases, the patient initially underwent superselective embolization of the pancreaticoduodenal artery using 350-560 μm gelatine sponge particles until near-complete flow stasis (**Figures 4A, B**). Following recurrent hemorrhage, a second superselective embolization was performed using gelatine sponge particles of 350-560 μm and 560-710 μm combined with three 0.018-inch Cook microcoils (3 mm diameter, 14 cm length; Cook Medical, Bloomington, IN, USA) to target the superior pancreaticoduodenal artery and gastroduodenal artery for durable occlusion (**Figures 4C, D**). In late November 2024, the patient was readmitted because of massive gastrointestinal bleeding. Endoscopy revealed progression of a large duodenal lesion with active bleeding and partial duodenal obstruction (**Figure 3D**), whereas CECT demonstrated progression of the caudate lobe lesion and a descending duodenal mass (**Figures 3F, G**). After multidisciplinary team consultation and endoscopic biopsy confirmation, an emergency third embolization was carried out using blank embolic microspheres (100-300 μm) plus a temperature-sensitive liquid embolic agent (TempSLE^®^; Beijing Guanhe medical technology) that solidifies at body temperature, achieving complete devascularization of the distal vascular bed (**Figures 4E, F**). Postoperative histopathological examination confirmed metastatic HCC; the immunohistochemical staining results revealed that the tumor tissue stained positive for Arg-1 (**Figures 4G, H**), a marker of primary HCC. As baseline for mRECIST assessment of the metastatic duodenal lesion, the initial CECT in April 2024 was used as the reference for target lesion selection; the follow-up CECT in March 2025 after two cycles of tislelizumab showed near-complete regression of viable components, consistent with a complete response (CR) by mRECIST criteria.

**Figure 4:**
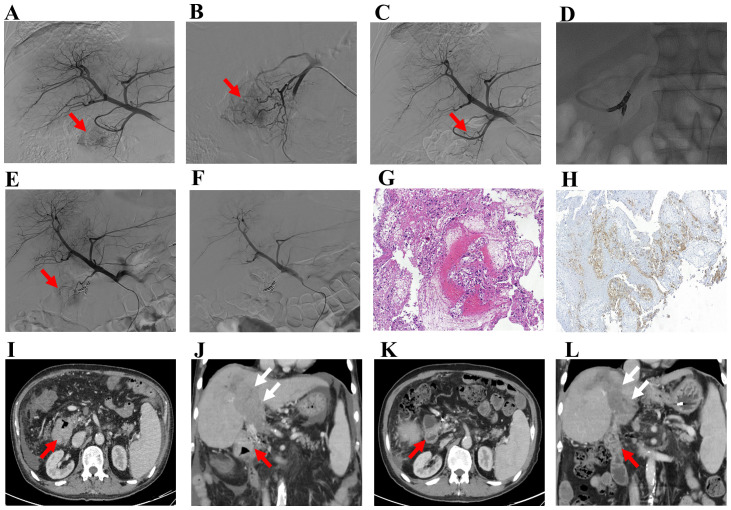
Three interventional embolization treatments, pathological diagnosis, radiological response following combined hemostasis and immunotherapy. **(A, B)** April 2024,first hemorrhage treated with embolization of the superior pancreaticoduodenal artery using gelatin sponge particles; **(C, D)** June 2024, second embolization, embolization of the gastroduodenal artery using gelatin sponge particles and coils; **(E, F)** November 2024, third embolization, recanalization of the pancreaticoduodenal artery, embolization with microspheres combined with liquid embolic agent, resulting in almost complete disappearance of tumor staining; **(G, H)** Histopathological examination of the duodenal biopsy specimen: **(G)** Hematoxylin and eosin (H&E) staining reveals nests of atypical epithelial cells; **(H)** Immunohistochemical staining is positive for Arginase-1 (Arg-1), confirming the diagnosis of metastatic hepatocellular carcinoma; **(I, J)** Baseline CT in November 2024 prior to immunotherapy: Portal vein tumor thrombus (white arrow in J); Marked thickening of the descending duodenal wall (red arrow in I, J); **(K, L)** Follow-up CT in March 2025 after two cycles of tislelizumab: Near-complete response of the portal vein tumor thrombus (white arrow in L); **(L)** Normalization of the previously thickened duodenal wall (red arrow in K, L).

Achieving hemostasis provides a pivotal therapeutic window that enables the timely initiation of subsequent antitumor treatment regimens. In January 2025, the patient underwent immunotherapy with tislelizumab (a PD-1 inhibitor). After two cycles of immunotherapy combined with the 12th session of HAIC, follow-up CECT in March 2025 revealed significant shrinkage of the primary caudate lobe lesion and PVTT compared to November 2024, as well as near-normalization of the descending duodenal wall thickness (**Figures 4I–L**). As of the latest follow-up (December, 2025), the patient had no recurrence of gastrointestinal bleeding. Repeat endoscopy was not performed after controlling the bleeding, as the patient had no recurrent symptoms, and follow-up CECT demonstrated near-normalization of the duodenal wall.

## Discussion

HCC with duodenal metastasis is an exceedingly rare clinical occurrence. The underlying metastatic mechanisms are hypothesized to involve direct invasion (especially from anatomically contiguous hepatic segments, such as the right hepatic lobe and caudate lobe), hematogenous dissemination, or lymphatic spread (6). Notably, prior locoregional therapeutic interventions, such as TACE, may induce tumor necrosis and local inflammation, which can promote tumor adhesion and facilitate tumor invasion into neighboring organs (7). In this case, the patient exhibited a caudate lobe hepatic lesion concurrent with extensive PVTT, a recognized high-risk factor for hematogenous metastasis. Furthermore, the patient’s history of multiple previous interventional procedures is consistent with the aforementioned pathophysiological mechanisms. A central feature of this case was the development and progression of duodenal metastasis, which posed diagnostic and therapeutic challenges.

The diagnosis of duodenal metastasis from HCC is frequently delayed, as initial manifestations, most commonly gastrointestinal bleeding, are often misattributed to portal hypertension in cirrhosis. In this case, the >2-year diagnostic delay resulted from a confluence of factors: bleeding was initially attributed to common variceal rupture, early endoscopic changes were subtle (mucosal elevation without mass, **Figure 3A**) and invisible on CECT (**Figure 3E**), and duodenal metastasis from HCC is exceptionally rare (<1%). A key clinical lesson is that systematic duodenal surveillance and a low biopsy threshold for any suspicious mucosa—even without active bleeding—could facilitate earlier diagnosis. These findings underscore the importance of maintaining a high index of clinical suspicion for gastrointestinal metastasis in HCC patients with high-risk features such as PVTT, particularly when atypical bleeding occurs. CECT serves as an essential noninvasive modality for diagnostic evaluation. In CT images, duodenal metastatic lesions typically demonstrate marked wall thickening with pronounced enhancement, which aligns with the hypervascular characteristics of HCC (8). A significant finding in this case was the superior sensitivity of endoscopy to that of CECT for early detection (**Figure 3E**), emphasizing the value of direct visualization when clinical suspicion is high. Multidisciplinary collaboration was crucial in the management of this complex case. The pivotal treatment strategy consisted of endoscopic biopsy followed immediately by emergency transarterial embolization (TAE). This integrated approach not only confirmed a pathological diagnosis but also concurrently mitigated the life-threatening risk of hemorrhage associated with the biopsy procedure.

At present, no standardized treatment protocol has been established for patients with HCC-derived duodenal metastasis presenting with bleeding. Although surgical resection is a potentially curative approach (9), it is frequently not feasible owing to compromised liver reserve and advanced tumor stage in these patients. For patients with gastrointestinal bleeding that is refractory to endoscopic therapy, TAE serves as an effective salvage therapy (10). In this case, the combination of a temperature-sensitive liquid embolic agent and embolic microspheres resulted in complete devascularization of the distal vasculature. This approach successfully controlled acute hemorrhage and, crucially, established a critical therapeutic window that facilitated the subsequent administration of systemic immunotherapy, ultimately leading to a significant clinical response.

The duodenal metastasis was first selectable as a target lesion in November 2024 when CECT showed measurable thickening with arterial enhancement. By mRECIST, this finding was interpreted as progressive disease (PD) relative to the prior unmeasurable state. After tislelizumab plus the 12th cycle of HAIC, the March 2025 CECT showed complete regression of viable enhancement, consistent with a CR by mRECIST.

Although this case report is hypothesis-generating and does not provide direct mechanistic evidence, multiple lines of preclinical evidence support a biologically plausible synergy between transarterial embolization (TAE) and PD-1 blockade: (i) hepatic artery embolization upregulates intratumoral and peritumoral PD-L1 expression (11); (ii) TAE modulates the immune microenvironment in both target and non-target HCC lesions (12); and (iii) microparticle TACE reshapes peripheral blood and tumor immune components (13). These observations collectively provide a strong rationale for the following hypothesis-driven discussion.

A particularly encouraging aspect of this case is the notable radiographic regression observed in both the primary hepatic lesion and the duodenal metastasis following hemostasis and combination therapy with tislelizumab. We postulate that this profound response is likely attributed to a synergistic, immune-mediated mechanism. Transarterial embolization is likely to induce localized tumor ischemia and necrosis, leading to the release of tumor-associated antigens and alterations in the local immune microenvironment (14). Concurrently, the resulting hypoxic environment may upregulate the expression of immune checkpoints, such as PD-L1, on tumor cells (15). The subsequent administration of a PD-1 inhibitor may have synergistically enhanced the systemic antitumor immune response. Drawing on these preclinical findings, we hypothesize that the synergy between TAE and PD-1 blockade may have contributed to the control of the embolized duodenal lesion and may have induced an abscopal-type response, which could at least in part explain the radiographic regression observed in the non-embolized primary tumor and portal vein tumor thrombus (16, 17). These observations suggest that a strategy integrating localized interventional therapy with systemic immunotherapy may represent a novel and promising therapeutic avenue for the management of advanced HCC with rare metastatic patterns.

The patient remained alive and clinically well at the last follow-up (December 2025). From initial diagnosis (September 2021) to last follow-up, overall survival reached at least 51 months. Quality of life improved markedly after bleeding control: body weight increased from 56 kg to 62 kg over three months, the patient recovered independent ambulation and light housework, and he consistently stated that he felt “much better than before”. No formal performance status (ECOG/KPS) was recorded, but the functional recovery and subjective well-being indicate a clinically meaningful benefit.

## Conclusion

This case demonstrates that multidisciplinary comprehensive management, centered on interventional embolization, is critical for addressing rare but life-threatening hemorrhage from hepatocellular carcinoma metastasized to the duodenum. Successful interventional hemostasis provides a crucial window for subsequent immunotherapy, and the potential synergistic antitumor effect of this combined approach may offer promising opportunities for achieving long-term survival with good quality of life in advanced hepatocellular carcinoma patients.

## Data Availability

The original contributions presented in the study are included in the article/supplementary material. Further inquiries can be directed to the corresponding author.

## References

[1] Sung H Ferlay J Siegel RL Laversanne M Soerjomataram I Jemal A . Global cancer statistics 2020: Globocan estimates of incidence and mortality worldwide for 36 cancers in 185 countries. CA Cancer J Clin. (2021) 71:209–49. doi: 10.3322/caac.21660. PMID: 33538338

[2] Kulik L El-Serag HB . Epidemiology and management of hepatocellular carcinoma. Gastroenterology. (2018) 156(2):477–91. doi: 10.1053/j.gastro.2018.08.065. PMID: 30367835 PMC6340716

[3] Reig M Sanduzzi-Zamparelli M Forner A Rimola J Ferrer-Fàbrega J Burrel M . Bclc strategy for prognosis prediction and treatment recommendations: The 2025 update. J Hepatol. (2025) 84(3):631–54. doi: 10.1016/j.jhep.2025.10.020. PMID: 41151697

[4] Liang J-D Chen C-H Hsu S-J Sheu J-C Yang P-M Lee H-S . Hepatocellular carcinoma with duodenal invasion and metastasis. J Gastroenterol Hepatol. (2012) 27:677–83. doi: 10.1111/j.1440-1746.2011.06869.x. PMID: 21793909

[5] Navuluri R Patel J Kang L . Role of interventional radiology in the emergent management of acute upper gastrointestinal bleeding. Semin Intervent Radiol. (2012) 29:169–77. doi: 10.1055/s-0032-1326925. PMID: 23997408 PMC3577584

[6] Lin CP Cheng JS Lai KH Lo GH Hsu PI Chan HH . Gastrointestinal metastasis in hepatocellular carcinoma: Radiological and endoscopic studies of 11 cases. J Gastroenterol Hepatol. (2000) 15:536–41. doi: 10.1046/j.1440-1746.2000.02152.x. PMID: 10847441

[7] Deforche M Bucalau A-M Tancredi I Tannouri F Verset G . Duodenal hemorrhage due to an invasive hepatocellular carcinoma controlled by transarterial embolization. Cureus. (2022) 14:e32046. doi: 10.7759/cureus.32046. PMID: 36600825 PMC9800851

[8] Park M-S Kim KW Yu J-S Kim M-J Yoon S-W Chung K-W . Radiologic findings of gastrointestinal tract involvement in hepatocellular carcinoma. J Comput Assist Tomogr. (2002) 26(1):95–101. doi: 10.1097/00004728-200201000-00014. PMID: 11801910

[9] Kato Y Matsubara K Akiyama Y Hattori H Hirata A Yamamoto T . Direct biliopancreatoduodenal invasion by hepatocellular carcinoma: Report of the first resected case and review of the literature. Int J Clin Oncol. (2010) 16:421–7. doi: 10.1007/s10147-010-0136-8. PMID: 20963617

[10] Zandrino F Tettoni SM Gallesio I Summa M . Emergency arterial embolization of upper gastrointestinal and jejunal tumors: An analysis of 12 patients with severe bleeding. Diagn Interv Imaging. (2016) 98:51–6. doi: 10.1016/j.diii.2016.04.003. PMID: 27178757

[11] Takaki H Hirata Y Ueshima E Kodama H Matsumoto S Wada R . Hepatic artery embolization enhances expression of programmed cell death 1 ligand 1 in an orthotopic rat hepatocellular carcinoma model: *In vivo* and *in vitro* experimentation. J Vasc Interv Radiol. (2020) 31(9):1475–82.e2. doi: 10.1016/j.jvir.2020.03.023. PMID: 32800663

[12] Tischfield DJ Gurevich A Johnson O Gatmaytan I Nadolski GJ Soulen MC . Transarterial embolization modulates the immune response within target and nontarget hepatocellular carcinomas in a rat model. Radiology. (2022) 303:215–25. doi: 10.1148/radiol.211028. PMID: 35014906 PMC8962821

[13] Ren Z Wang Y Jiang D Liu Y Yang X Wang T . Pd1+ Treg cell remodeling promotes immune homeostasis within peripheral blood and tumor microenvironment after microparticles-transarterial chemoembolization in hepatocellular carcinoma. Cancer Immunol Immunother. (2025) 74:109. doi: 10.1007/s00262-025-03962-z. PMID: 39937280 PMC11822157

[14] Llovet JM De Baere T Kulik L Haber PK Greten TF Meyer T . Locoregional therapies in the era of molecular and immune treatments for hepatocellular carcinoma. Nat Rev Gastroenterol Hepatol. (2021) 18:293–313. doi: 10.1038/s41575-020-00395-0. PMID: 33510460

[15] Tan J Fan W Liu T Zhu B Liu Y Wang S . Trem2+ macrophages suppress Cd8+ T-cell infiltration after transarterial chemoembolisation in hepatocellular carcinoma. J Hepatol. (2023) 79:126–40. doi: 10.1016/j.jhep.2023.02.032. PMID: 36889359

[16] Shaha S Rodrigues D Mitragotri S . Locoregional drug delivery for cancer therapy: Preclinical progress and clinical translation. J Control Release. (2024) 367:737–67. doi: 10.1016/j.jconrel.2024.01.072. PMID: 38325716

[17] Cao Y Xiong F Kan X Guo X Ouyang T Wang R . Transarterial viroembolization improves the therapeutic efficacy of immune-excluded liver cancer: Three birds with one stone. Pharmacol Res. (2022) 187:106581. doi: 10.1016/j.phrs.2022.106581. PMID: 36436709

